# The role of lipid metabolism in cognitive impairment

**DOI:** 10.1055/s-0044-1792097

**Published:** 2025-01-15

**Authors:** Meifang Xu, Liyuan Wang, Yun Meng, Guiqiong Kang, Qing Jiang, Tao Yan, Fengyuan Che

**Affiliations:** 1 Second Medical University, School of Clinical Medicine, Weifang Shandong Province, China.; 2 Linyi People's Hospital, Department of Neurology, Linyi Shandong Province, China.; 3 Guangzhou University of Chinese Medicine, Linyi People's Hospital, Linyi Shandong Province, China.; 4 Harbin Medical University, First Affiliated Hospital, Department of Neurosurgery, Harbin Heilongjiang Province, China.; 5 Key Colleges and Universities, Laboratory of Neurosurgery, Harbin Heilongjiang Province, China.

**Keywords:** Cognitive Dysfunction, Lipid Metabolism, Apolipoproteins E

## Abstract

Alzheimer's disease (AD), diabetic cognitive impairment (DCI), and vascular dementia (VD) are considered the most common causes of severe cognitive impairment in clinical practice. Numerous factors can influence their progression, and many studies have recently revealed that metabolic disorders play crucial roles in the progression of cognitive impairment. Mounting evidence indicate that the regulation of lipid metabolism is a major factor in maintaining brain homeostasis. Generally, abnormalities in lipid metabolism can affect amyloid-beta (Aβ) deposition, tau hyperphosphorylation, and insulin resistance through lipid metabolic signaling cascades; affect the neuronal membrane structure, neurotransmitter synthesis and release; and promote synapse growth, which can impact neural signal transmission and exacerbate disease progression in individuals with cognitive impairment, including AD, DCI, and VD. Moreover, apolipoprotein E (APOE), a key protein in lipid transport, is involved in the occurrence and development of the aforementioned diseases by regulating lipid metabolism. The present article mainly discusses how lipid metabolic disorders in the brain microenvironment are involved in regulating the progression of cognitive impairment, and it explores the regulatory effects of targeting the key lipid transport protein APOE in the context of the role of lipid metabolism in the common pathogenesis of three diseases—Aβ deposition, tau hyperphosphorylation, and insulin resistance—which will help elucidate the potential of targeting lipid metabolism for the treatment of cognitive impairment.

## INTRODUCTION

Cognitive impairment, especially severe cognitive impairment (dementia), has resulted in substantial social and economic burdens worldwide. Currently, the global age-standardized prevalence of dementia in people aged 60 years or older has reached 5 to 7%, and the prevalence is increasing annually, with a projected doubling in approximately 5 years. It is estimated that there will be more than 65.7 million dementia patients worldwide by 2030, and this number may reach 115.4 million by 2050. 1 China is the country with the largest population with dementia in the world, and epidemiological surveys conducted in 2020 2 indicate that there are around 15.07 million dementia patients over 60 years of age in China, of which approximately 9.83 million suffer from Alzheimer's disease (AD; ∼ 65% of all dementia patients), and vascular dementia (VD) accounts for ∼ 15 to 20% of all dementia patients. Therefore, the prevention and treatment of dementia remain challenging for society.

Alzheimer's disease, diabetic cognitive impairment (DCI), and vascular dementia (VD) are common causes of severe cognitive impairment in the clinical practice. 3 Studies have been increasingly reporting that these three types of dementia present similar pathogeneses, 4 including amyloid-beta (Aβ) protein deposition, tau protein hyperphosphorylation and insulin resistance. Moreover, studies have confirmed that lipid metabolism disorders are closely related to these three types of cognitive impairment progression. A meta-analysis, for example, confirmed that a high serum cholesterol level in middle-aged people is an important risk factor for AD pathology. 5 Although research on DCI is lacking, recent studies 6 have confirmed that lipid droplet accumulation in microglia can affect the occurrence and progression of DCI. Traditional vascular risk factors—such as diabetes, hypercholesterolemia, hypertension, and smoking—are considered risk factors for VD, and increasing evidence suggest that hypercholesterolemia, a lipid metabolic disorder, may be a major pathogenic factor for VD. 7 Alzheimer's disease is the most common neurodegenerative disease, and neurons express receptors for various adipokines, suggesting that factors released by adipose tissue have the potential of communicating directly with the brain. Research confirm that the metabolic changes and increased inflammatory state associated with obesity can lead to damage to the central nervous system, which can result in nerve death and altered neuronal synaptic plasticity; this metabolic dysfunction increases the risk of cognitive dysfunction. 8

Although a large body of epidemiological and clinical evidence 9 have shown that lipid metabolic abnormalities are independent risk factors for dementia development, the underlying relationship between lipid metabolic abnormalities and the risk of dementia remains to be revealed. In the present review, we summarize and discuss the recent progress in elucidating the relationships involving lipid metabolism and AD, DCI, and VD, aiming to explore the potential role of lipid metabolism in the pathogenesis of these types of dementia.

## LIPID METABOLISM AND COGNITIVE IMPAIRMENT

Due to its latent involvement in the progression of dementia, abnormal lipid metabolism has received increasing attention in recent years. Mounting evidence suggest that lipid metabolism plays a prominent role in the occurrence and development of dementia. 10 A study by Anand et al. 11 linked lipid metabolism and cognitive impairment even more directly. Their study 11 suggests that an excess of lipids contributes to lower cognitive scores by increasing risk factors such as cardiovascular risk, which may be associated with vascular brain damage, as well as cognitive impairment.

During signal transmission in the central nervous system (CNS), a series of physiological processes, including cell membrane composition, myelin formation, synaptic formation, and the synthesis and release of neurotransmitters, are closely associated with lipid metabolic regulation. Glycolipids, consisting of hydrophobic lipid tails and one or more sugar groups linked by glycosidic bonds, are found on the outer leaflets of eukaryotic cell membranes, where they confer membrane stability and facilitate intercellular communication and signal transduction. Cholesterol integrally alters the permeability-barrier properties of cell membranes by reducing the deformability and fluidity of the lipid bilayer. 12 Accordingly, abnormal lipid metabolism interferes with neural signal transmission, thereby negatively affecting cognitive function. 13 Lipids are important components of cell membranes and are essential for maintaining the normal structure and function of neurons. Cholesterol, an important lipid, is involved in the formation of specialized membrane microdomains, such as lipid rafts and ion pumps, which separate cellular processes. These microdomains are formed through interactions among enriched cholesterol, sphingolipids, proteins, and other substances. 14 Lipid rafts contain more cholesterol and saturated fat than the surrounding lipid bilayer membrane structure; thus, they have greater fluidity. Therefore, lipid rafts provide a tight structural framework to signal molecules and other proteins on the cell surface, enabling lipid rafts to play roles in signal transduction and protein sorting. In addition, cholesterol is involved in the regulation of ion channel function through interactions with sphingolipids. 15

Lipid metabolism is also involved in the synthesis and metabolism of various bioactive substances in the brain, including neurotransmitters and hormones, and these substances play important roles in cognitive processes. 16 Imbalances in these bioactive substances caused by abnormal lipid metabolism can lead to demyelination and axonal loss, which further affect cognitive function. 17 Moreover, lipids regulate neuronal growth and development; synaptic plasticity; axonal growth, extension, and regeneration; and dendritic growth. Sphingomyelin, galactosylceramide, and sulfide, for example, are highly enriched in oligodendrocytes, which can promote the wrapping of myelin sheaths around axons to accelerate neural transmission and support neuronal function. 10

**Figure 1:**
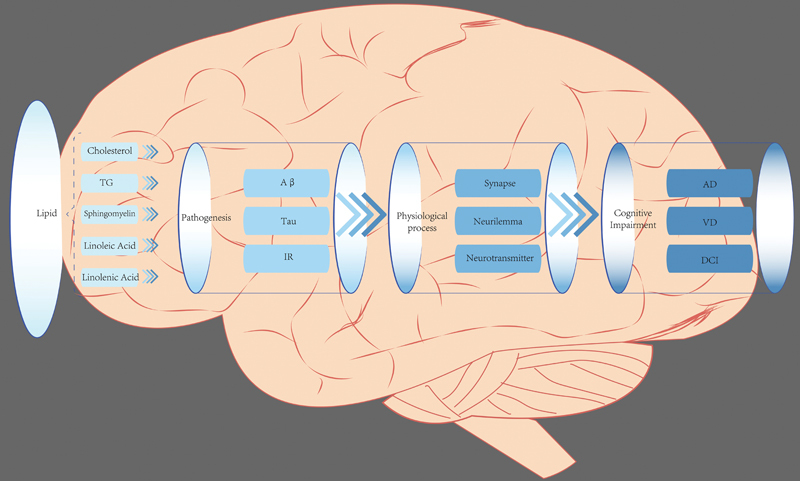
The crucial roles of lipid metabolism in cognitive impairment.

In conclusion, abnormal lipid metabolism can interfere with neural signaling and affect cognitive function by causing changes in the structure of the neuronal membrane, affecting neurotransmitter synthesis and release, and influencing synapse formation and extension ( Figure 1 ). Therefore, the role of lipid metabolism in cognitive impairment needs further investigation. We herein review the role of abnormal lipid metabolism in AD, DCI, and VD.

### Lipid metabolism and AD

Critical histopathological changes, including the extracellular accumulation of neuritic plaques (NPSs, also known as senile plaques) and the intracellular accumulation of neurofibrillary tangles (NFTs), substantially contribute to synaptic defects and neurodegeneration in AD. 18 Previous studies 19 have shown that an imbalance in lipid metabolism may play an important role in AD progression. Toxic lipid accumulation occurs when the metabolic balance of lipids is disrupted, resulting in membrane protein injury, subsequently causing notable damage to neurons and synaptogenesis and, ultimately, leading to cognitive impairment. 19

In addition, the cellular energy supply, signaling, and senile plaque clearance are influenced by lipid metabolism. Normalizing lipid metabolism in microglia, for example, has been shown to increase the production of adenosine triphosphate (ATP), which, in turn, promotes Aβ clearance in AD. Lipids, especially cholesterol, account for a very high proportion of brain weight. As a major lipid required to maintain cell membrane homeostasis, the amount of cholesterol in the membrane bilayer influences the permeability and fluidity of cell membranes and, more importantly, cholesterol is involved in cellular signaling processes such as synapse formation, intracellular protein sorting and transport, synaptic plasticity, and neuronal degeneration. 20 With lipid metabolism being studied more intensively, altered cholesterol metabolism has been found in AD. 21 Since cholesterol is a major component of neuronal membranes, it plays an important role in maintaining the metabolism and homeostasis of various membrane proteins, such as the amyloid precursor protein (APP) and the acetylcholine receptor (ACHR). 22 Moreover, cholesterol deficiency can increase the susceptibility of hippocampal glia in primary culture to glutamate-induced excitotoxicity, affecting the synthesis of neurosteroids and changing the fluidity of cell membranes, ultimately altering the physicochemical properties of cell membranes in AD. 23

Lipid rafts are specialized cholesterol-enriched microdomains in the cell membrane. Overall, the cell membrane is a united system of opposites composed of many different microregions that are relatively independent at the microscopic level and absolutely linked at the macroscopic level, and lipid rafts, that is, microstructural domains on the surface of the membrane, present an asymmetry of membrane lipids. Lipid rafts serve as hubs to aggregate proteins, such as neurotransmitter receptors, ion channels, and synaptic proteins, 24 which can act as anchors for β-secretase and gamma-secretase (γ-secretase), which are directly related to the production of the Aβ protein. 25 Hence, the regulation of lipid rafts by maintaining the balance of membrane proteins and the fluidity of cholesterol in cell membranes affects the progression of AD. In addition, cholesterol, as a constituent of synapses, can influence neural signal transmission and disrupt the homeostasis of neurons, which consequently results in the malfunction of neural synapses and neuronal death.

Additionally, cholesterol can influence AD progression by regulating the extent of Aβ fibrilization as well as the clearance of Aβ peptides. 26 Cholesterol levels are positively correlated with the degree of Aβ precursor protein (AβPP) processing, and the levels of cholesteryl esters and free cholesterol can affect amyloid formation. 27 In conclusion, increasing cholesterol levels effectively prevent or reverse pathological changes associated with AD, including Aβ deposition, synaptic injury, autophagic inhibition, and apoptosis.

Besides cholesterol, other lipids are equally important in AD progression. Phospholipids are closely related to the regulation of neuronal dendritic branching, synapse growth, and synaptic vesicle shuttling. 10 Glycerophospholipids and sphingomyelins are two types of phospholipids that are basic components of cell membranes. Sphingomyelins can combine with cholesterol to promote the construction of lipid rafts, which are considered crucial membrane components. 28 Phosphatidylinositol phosphates (PtdInsPs) are phosphorylated derivatives of phosphatidylinositol (PI) that are docking sites for actin cytoskeleton regulatory factors (Wiskott–Aldrich syndrome proteins). 29 Phosphatidylinositol phosphates can bind to lipid-binding proteins (such as bin-amphiphysin-Rvs, BAR), which further interact with actin regulatory factors to promote neural bud branching. 30 Although phosphatidic acid (PA) comprises only a small part of the cell membrane, this molecule supports neurite elongation by stimulating vesicle fusion to promote membrane expansion. 31 Sphingolipids are found primarily in neurons and are highly enriched in synapses. 32 Thus, neurogenesis, the process by which neural stem cells (NSCs) differentiate into mature neurons and glia, cannot occur without the involvement of gangliosides and other sphingolipids. 33 For example, depletion of GD3 (a small ganglioside converted from GM3 ganglioside by α-2,8-sialyltransferase [GD3 synthase]) impairs neurogenesis and reduces dendrite complexity and spine density. 34 Furthermore, GM1 interacts with neurotrophic factors and their receptors, further promoting neuronal growth and survival. 35 Consequently, these lipids can participate in the regulation of the structure of the neuronal membrane, synapse formation, and neuronal survival during the progression of AD, ultimately having a profound impact on the prognosis of AD patients.

### Lipid metabolism and DCI

Cognitive impairment is a catastrophic injury to the CNS in patients with type-2 diabetes mellitus (T2DM) that seriously affects their quality of life and imposes a major economic burden on society. Patients with DCI have lipid metabolic abnormalities. Obesity and dyslipidemia are major risk factors for cognitive dysfunction in diabetic patients. Some studies 36 have shown that cholesterol metabolism is strongly associated with neurodegenerative diseases. Previous studies 37 have revealed that several risk factors for AD involve genes for lipid metabolism and transport, including *APOE4* , *CLU* , and *ABCA7* ; therefore, abnormal lipid metabolism may be important in DCI. Multiple signaling pathways involving inflammatory responses, oxidative stress, and mitochondrial dysfunction have been shown to affect cognitive function in diabetic patients. 38 In healthy individuals, insulin can inhibit the release of free fatty acids by inhibiting lipolysis. However, in diabetic patients, insulin resistance interferes with the regulation of lipolysis by insulin, resulting in increased levels of free fatty acids in the blood and increased Aβ protein deposition. The overload of free fatty acids in the blood also leads to lipotoxicity, which further exacerbates insulin resistance, ultimately creating a vicious cycle. 39

Moreover, small-vessel disease caused by endothelial cell injury is a common complication in patients with DCI. Abnormal lipid metabolism leads to elevated blood lipid levels, and the accumulation of lipids in blood vessels damages endothelial cells, causing cerebrovascular disease and affecting cerebral blood flow and neuronal function, which ultimately exacerbates the progression of disease in patients with DCI. 40 Additionally, patients with DCI often exhibit neuroinflammation, which is involved in vascular damage, neurodegeneration, and pathological Aβ changes. These pathological changes can exacerbate the progression of cognitive impairment in patients with DCI. Research has shown that lipid metabolism is closely related to neuroinflammation. Phosphoinositide (PI) 3-kinase (PI3K), for example, is an important inflammatory cell signaling molecule that can phosphorylate the 3-position hydroxyl group of PIs. Lipid second messengers, including PtdInsPs and lactosylceramide, directly or indirectly participate in the PI3K signaling process. Specifically, phosphatidylinositol bisphosphate (PIP2), a substrate of PI3K, can activate the downstream Akt pathway, 41 whereas lactosylceramide can induce the activation of proinflammatory microglia, ultimately leading to the secretion of proinflammatory cytokines and inducible nitric oxide synthase. 42 Thus, abnormal lipid metabolism can exacerbate disease progression in patients with DCI by inducing neuroinflammation.

### Lipid metabolism and VD

Cognitive impairment caused by cerebrovascular disease has been termed *vascular cognitive impairment* (VCI). Since atherosclerotic vascular disease is the most likely cause of VD, lipid metabolism analysis and dyslipidemia have become top research priorities. 37 Research has shown 43 that lipid metabolism also plays an important role in the occurrence and development of VCI.

Middle cerebral artery atherosclerosis (MCAA) is the main causative mechanism of VD, and it leads to narrowing or occlusion (complete blockage) of blood vessel lumens. Lipid metabolic disorders may lead to elevated levels of cholesterol, triglycerides, and other lipids in the blood that accumulate in vessel walls, 44 thereby triggering cerebral arteriosclerosis. Arteriosclerosis can cause insufficient blood supply to brain tissue, followed by ischemia and hypoxia in brain tissue due to impaired blood supply to the brain, resulting in necrosis, softening, and, eventually, infarction. 45

Moreover, lipid metabolism is closely related to cerebral white matter status. In white matter, the oligodendrocyte membrane composed of lipids wraps the axons of neurons to form the myelin sheath, preventing nerve damage, which plays an important role in cognitive function. Mounting evidence has confirmed 46 that white matter rarefaction is another important factor in the progression of VD, and the results of fluid-attenuated inversion recovery (FLAIR) or T2-weighted magnetic resonance imaging (MRI) scans have shown that rarefaction and calcification of cerebral white matter in patients with VD increase with age. White matter hyperintensity (WMH) is not only a typical imaging feature of cerebral small-vessel disease (CSVD), but it is also an important biomarker of VCI, whereas evidence 47 suggests that lipoatrophy and atherosclerosis are important causes of cerebral WMH. Thus, lipid metabolic abnormalities may be involved in the progression of VD by affecting the degeneration of cerebral white matter.

Increased blood-brain barrier (BBB) permeability can also exacerbate the progression of VD. An increase in toxic lipids can lead to endothelial dysfunction, thereby increasing the levels of inflammatory mediators such as interleukin-1 (IL-1) and subsequently damaging the BBB. Destruction of the BBB exposes the brain parenchyma to neurotoxic blood proteins, thrombin, fibrinogen, prothrombin, and hemoglobin, leading to abnormal neuronal activity and accelerating the progression of VD. Therefore, abnormalities in lipid metabolism play an important role in the pathogenesis of VD and may provide a viable option for lipid metabolism-targeted therapy for VD.

## LIPID METABOLISM AND THE PATHOGENESIS OF COGNITIVE IMPAIRMENT

**Figure 2:**
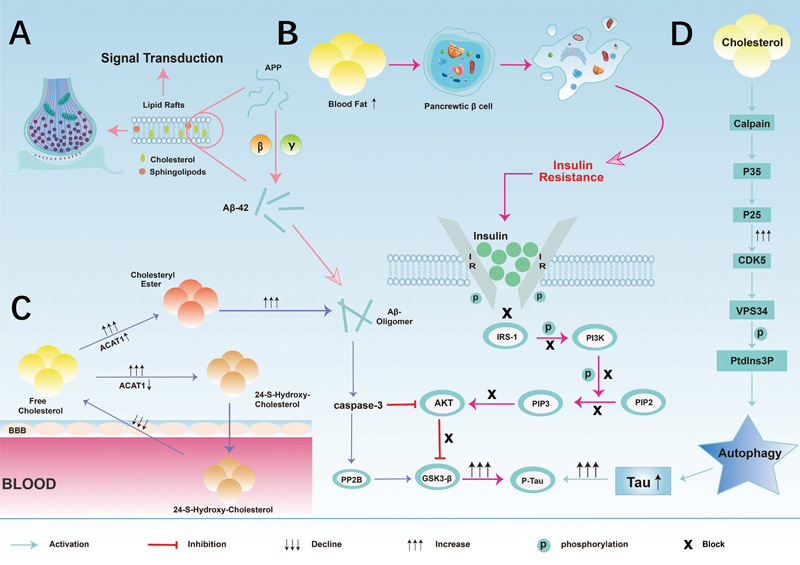
The relationship between lipid metabolism and cognitive impairment. ( **A** ) Association of lipid rafts with amyloid-beta (Aβ) deposition and tau protein phosphorylation. ( **B** ) High-fat leads to insulin resistance and phosphorylated tau accumulation. ( **C** ) Free cholesterol affects Aβ protein deposition and phosphorylated tau accumulation via the acyl-coenzyme A cholesterol acyltransferase 1 (ACAT1)-signaling pathway. ( **D** ) Cholesterol affects phosphorylated tau accumulation by modulating the autophagy pathway.

Deposition of the Aβ protein, tau protein hyperphosphorylation, and insulin resistance 4 have been research hotspots in the field of cognitive disorders, and these pathogenic mechanisms coexist in AD, DCI, and VD. However, the close correlation between lipid metabolism and the common pathological mechanisms of these conditions has attracted the attention of an increasing number of scholars ( Figure 2 ). In this section, we summarize the biological features of lipid metabolism to provide a comprehensive overview of the intrinsic mechanisms by which lipid metabolism regulates Aβ protein deposition, tau protein hyperphosphorylation, and insulin resistance and emphasize its important role in the pathogenesis and progression of AD, DCI and VD, providing novel insights and approaches for the prevention and treatment of these types of dementia.

### Lipid metabolism and Aβ deposition

A peptide consisting of 39-43 amino acids, Aβ is produced by the proteolytic action of β- and γ-secretases on APP. Research 48 suggests that Aβ protein deposition plays a fundamental role in the development of various conditions associated with cognitive impairment, such as AD, DCI, and VD. The cleavage of APP by secretases occurs in cholesterol-rich lipid rafts ( Figure 2A ). The Aβ peptides are hydrophobic, and their production and release in the membrane may be affected by the lipid composition of the membrane. Therefore, the membrane lipid composition plays an important role in the pathogenesis of cognitive impairment. Many studies 49 50 have shown that changes in cholesterol levels in the lipid bilayer structure can affect APP processing, leading to changes in Aβ production, suggesting that lipid metabolic abnormalities are closely related to Aβ protein deposition. In addition, tau accumulation induced by lipid metabolic abnormalities can mediate Aβ toxicity, which is largely dependent on the presence or absence of tau. The Aβ oligomers are formed by the hyperphosphorylation of tau proteins, leading to the aggregation of said proteins into oligomers, which evolve into paired helical filaments and, ultimately, tangles. Tau oligomers are also toxic to neurons and have a strong effect on synapses, which, in turn, induces the formation of Aβ oligomers. 51

In the brain, cholesterol accounts for a large part of the lipid content in cells, and it exists mainly in a nonesterified form in the myelin sheaths and cell membranes of glial cells and neurons. Due to the presence of the BBB, most cholesterol in the brain comes from self-synthesis within astrocyte cells. 52 Excessive free cholesterol in cells can be converted into cholesteryl esters by acyl-coenzyme A cholesterol acyltransferase 1 (ACAT1), after which the accumulated lipid droplets can be transported to the extracellular environment. 53 Increased cholesteryl ester levels promote the release of Aβ in cultured cells, whereas pharmacological inhibition of ACAT1 results in the conversion of excess free brain cholesterol into 24(S)-hydroxycholesterol, which crosses the BBB to the periphery, removing Aβ and cholesteryl esters ( Figure 2C ). Research 54 has also confirmed that inhibition of ACAT1 expression can reduce Aβ accumulation and improve cognitive impairment in AD mouse models. These data collectively indicate that the balance between free cholesterol and cholesteryl esters is a key regulatory link in controlling amyloid deposition, but the molecular mechanisms of this balance have rarely been reported and will be an important research direction in the future.

Several findings indicate that cholesterol efflux also controls Aβ production. Adenosine triphosphate-binding cassette transporter A1 (ABCA1) is an important regulator of apolipoprotein E (APOE) levels and lipidation status, and it can stimulate cholesterol efflux to reduce intracellular Aβ levels. In-vivo studies have shown 55 56 57 that the deletion of ABCA1 in AD mouse models significantly reduces the levels of APOE in the brain and periphery, leading to increased Aβ deposition. Furthermore, since the remaining APOE in ABCA1-deficient mice has a low degree of lipidation, the reduction in APOE lipidation, rather than the low levels of APOE, may suppress cholesterol efflux to accelerate amyloid formation. 58

Overconsumption of a high-fat diet (HFD), specifically a diet rich in the saturated fatty acid (SFA) known as palmitic acid (PA), can exacerbate neuroinflammation, neurodegeneration, and cognitive impairment. Increased free fatty acid levels can affect cell membrane permeability, redox potential, and even the processing of AβPP to increase Aβ deposition. 59

### Lipid metabolism and tau hyperphosphorylation

Microtubules are considered critical structures for stable neuronal morphology because they serve as tracks for long-distance transport, provide dynamic and mechanical functions, and control local signaling events. Thus, proteins that associate with principal cytoskeletal components, such as microtubules, strongly affect both the morphology and physiology of neurons. Tau, a microtubule-associated protein, can stabilize neuronal microtubules by interacting with lipids under normal physiological conditions. However, under certain pathological conditions, the overphosphorylation of tau proteins separates them from microtubule structures, increasing their susceptibility to form tangles, which is detrimental to the stability of microtubule structures. The overphosphorylation of tau proteins leads to an abnormal increase in the number of cytoskeletal proteins, impaired axoplasmic transport, and neuronal degeneration. 60 Lipid metabolic disorders are crucial regulators of tau protein phosphorylation. Research 61 has shown that lipid metabolic disorders can regulate tau protein hyperphosphorylation through various pathways; the Aβ signaling pathway, for example, can disrupt the biochemical pathways associated with lipid metabolic enzymes and bioactive lipids, thereby affecting the hyperphosphorylation of the tau protein. Cholesterol levels can regulate the hydrolysis of the tau protein by regulating the activity of calpain. In addition, lipid rafts, which are sterol- and sphingolipid-rich domains, maintain nervous system function in a variety of ways, including by modulating tau pathology. Lipid metabolic disorders can lead to changes in the structure and function of lipid rafts, thereby affecting the physiological and pathological processes of the tau protein. Indeed, lipid rafts contain a pool of hyperphosphorylated tau, indicating that cholesterol may modulate tauopathies. 62 Moreover, Aβ accelerates the phosphorylation of tau proteins by mediating tau phosphorylation-related kinases such as cell cyclin-dependent kinase 5 (CDK5) activator 1 and glycogen synthase kinase 3β (GSK3β). The specific mechanism involves CDK5 promoting the phosphorylation of the lipid kinase PtdIns 3-kinase catalytic subunit type 3 (PIK3C3, also known as VPS34), 63 which induces its product, PtdIns 3-phosphate (PtdIns3P) to regulate the clearance of tau aggregates through the stimulation of autophagy pathways 64 ( Figure 2D ). In addition to interfering with tau phosphorylation, Aβ interferes with tau oligomerization and aggregation, increasing neuronal damage through the activation of CDK-5- and GSK-3β-mediated tau oligomer formation, which, in turn, leads to neurodegeneration. 65 Therefore, lipid metabolic disorders may affect the activity of these kinases, ultimately affecting tau hyperphosphorylation. Besides the aforementioned endogenous factors, exogenous high cholesterol intake induces tau hyperphosphorylation, oligomerization, and aggregation to promote the progression of tau pathology.

Taken together, these findings indicate that lipid metabolic abnormalities play a key role in the hyperphosphorylation of tau protein and can affect the tau protein phosphorylation pathway through multiple pathways, ultimately affecting the progression of the disease.

### Lipid metabolism and insulin resistance

Insulin is a 51-amino acid peptide hormone secreted by pancreatic β-cells, and its core function is to maintain blood glucose within physiological ranges by promoting glucose uptake and inhibiting glucose production and release by the liver. However, insulin also has other functions; for example, it can act as an antimetabolic hormone to impact both the metabolic process of fat breakdown and the intake of fatty acids. Brain insulin resistance occurs when brain cells do not respond to insulin. 66 Mechanistically, brain insulin resistance is caused by either the downregulation of insulin receptors, which prevents them from binding to insulin, or the erroneous activation of insulin signaling cascades. Functionally, brain insulin resistance can manifest as impaired metabolic regulation of the brain or cognitive and affective impairments, and the specific mechanisms are related to a reduction in neuronal plasticity through the regulation of physiological processes related to insulin metabolism, including the regulation of neuronal receptor expression, the impairment of neurotransmitter release, and the induction of neuroinflammation. 67 Mechanistically, downregulation of insulin receptor (IRs) expression and impairment of insulin receptor substrate (IRS) proteins characterize insulin resistance. Insulin receptors are localized to pre- and postsynaptic neurons, and they recruit and activate the PI3K complex, which, in turn, activates Akt downstream of the Akt, glucose transporter type 4 (GLUT4), and mechanistic target of rapamycin (mTOR) complexes. The Akt-mediated stimulation of mTOR and its downstream targets regulates protein and lipid synthesis and promotes dendritic spine formation, neuronal development, survival, autophagy, and synaptic plasticity. 37

Increasing evidence 68 have shown that lipotoxicity caused by long-term hyperlipidemia can negatively affect the metabolism and secretion of pancreatic β-cells, causing cell death and exacerbating insulin resistance ( Figure 2B ). Additionally, insulin resistance is related to an increase in circulating nonesterified fatty acids, which can lead to elevated plasma cholesterol levels, ultimately leading to systemic inflammation and exacerbating the progression of cognitive impairment. 69 Normal adipocytes in adipose tissue can increase and maintain nerve cell sensitivity to insulin by secreting adipokines and other regulatory molecules, such as leptin and adiponectin. 70 Research 71 has also confirmed that in insulin-resistant patients with obesity, the adipose tissue secretes molecules that antagonize the effects of insulin, including cytokines and some proinflammatory factors, such as retinol-binding protein 4 (RBP4), tumor necrosis factor-alpha TNF-α, interleukin-6 (IL-6), and interleukin-1beta (IL-1β), and these molecules can stimulate adipose tissue proliferation and systemic inflammatory responses. In conclusion, abnormal lipid metabolism and insulin resistance can interact and form a vicious cycle, promoting pathological processes related to cognitive impairment.

## APOLIPOPROTEIN E AND THE PATHOGENESIS OF COGNITIVE IMPAIRMENT

**Figure 3:**
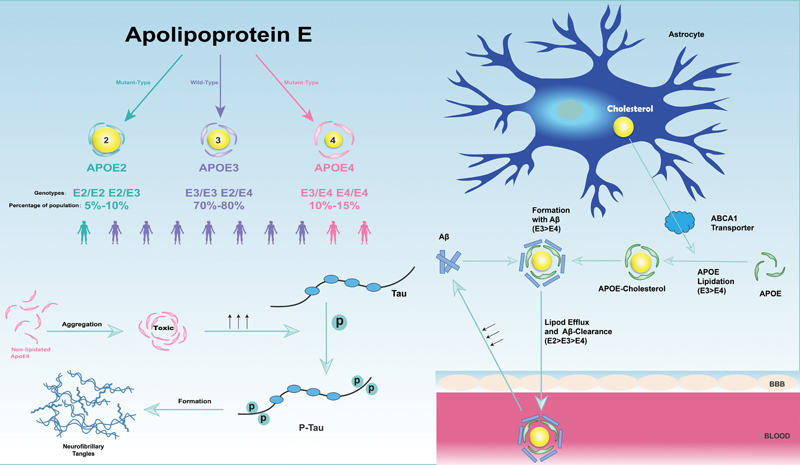
Complex interactions between APOE and the pathogenesis of cognitive impairment.

**Figure 4:**
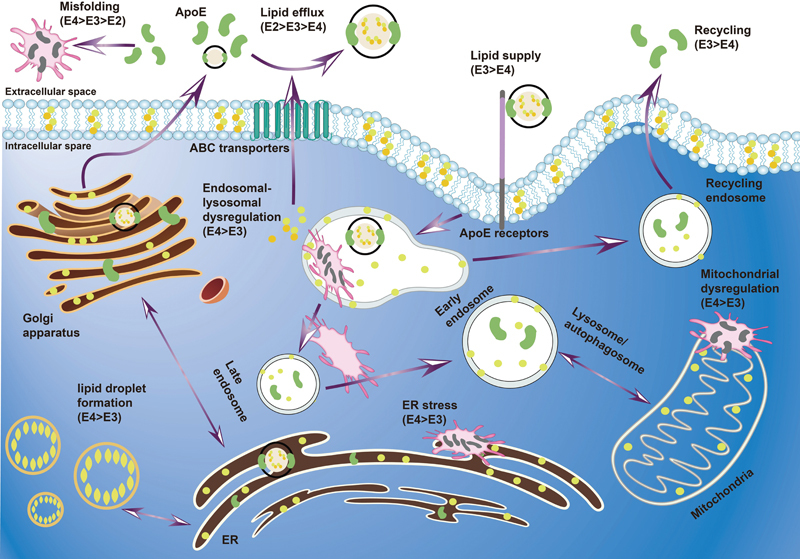
Apolipoproteins and cellular homeostasis. Note: extracted from Martens et al. 96

The balance between lipid efflux and endocytosis not only regulates the balance of lipids in the brain but also affects the health of neurons. The most abundant apolipoprotein, APOE is a vital regulator of lipid metabolism 72 ( Figure 3 ). Research has confirmed 73 that different APOE alleles have different functions in lipid metabolism, which leads to varying effects on neuroinflammation and lipoprotein composition ( Figure 4 ); therefore, APOE, especially APOE4, is widely considered to be associated with an increased risk of cognitive impairments. An earlier study 74 revealed differences between the isoforms: compared with APOE3, APOE4 inhibits synaptic plasticity in the hippocampus and internal olfactory cortex after environmental stimulation.

Given that APOE is a key molecule in lipid metabolism that plays an important role in the pathogenesis of cognitive impairment, in the present study, we aimed to explore the potential link between APOE-mediated lipid metabolic regulation and the shared pathological mechanisms of the three categories of cognitive impairment (AD, DCI, and VD), including Aβ protein deposition, tau protein hyperphosphorylation, and insulin resistance.

### Apolipoprotein E and Aβ

The regulation of Aβ deposition by APOE has been reported by multiple studies. Some studies have confirmed 75 that APOE is directly involved in the metabolic processing of APP/Aβ. Research 76 has also confirmed that the interaction between APOE and the pathological deposition of Aβ is an important mechanism by which APOE affects cognitive impairment, such as in AD, DCI, and VD. Reports 77 have shown that the originally-dense Aβ plaques become loose in mice with endogenous APOE knockout, indicating that APOE plays an important role in Aβ deposition. Interestingly, the effect of APOE on Aβ is isomer-dependent; for example, APOE2 and APOE4 have the greatest impact on Aβ deposition in patients relative to APOE3, which is the most common genotype in the population. Carriers of APOE2 accumulate less Aβ, whereas APOE4 carriers accumulate more Aβ. The risk of disease is increased 3- to 4-fold in patients with 1 APOE4 allele, whereas the risk is increased 20-fold in patients with 2 APOE4 alleles. Moreover, compared with those of other isoforms, the folding structural features of the APOE4 protein are more compact, which leads to a reduced ability to bind to Aβ complexes, increasing the likelihood that Aβ complexes will be cleaved into neurotoxic fragments. 78

Multiple subsequent studies have confirmed 79 80 that APOE4 overexpression could hinder the clearance of perivascular antibodies, and this decrease in antibody clearance capacity can lead to a decrease in the clearance capacity of Aβ peptides, which results in the development of amyloid plaques and other pathological features, including changes in the neurovascular unit and BBB function. Moreover, APOE4 is associated with elevated levels of Aβ40, a subtype of the Aβ protein, which is a characteristic type of amyloid deposition in cerebral amyloid angiopathy (CAA). These findings clearly indicate that APOE4 overexpression is closely linked to Aβ deposition. Hence, the different APOE subtypes have different effects on Aβ deposition, and the ability of APOE to clear Aβ protein deposition must be strictly controlled for genotyping in clinical treatments, which will provide insight into the interaction between APOE-mediated Aβ accumulation and lipid metabolic disorders.

Besides promoting Aβ plaque formation, APOE also is involved in Aβ clearance through various mechanisms, such as receptor-mediated clearance and proteolytic degradation. The low-density lipoprotein receptor-related protein 1 (LRP1) receptor in neurons mediates Aβ clearance through uptake of the Aβ/APOE complex. And because of the reduced stability of the APOE4/Aβ complex, the uptake process of Aβ is impaired in APOE4 carriers. which is further exacerbated by altered receptor binding and competition for Aβ receptor binding with APOE, resulting in significantly reduced receptor-mediated clearance in the presence of APOE4. In addition, soluble Aβ can be cleared by proteolytic enzymes such as metalloendopeptidases, fibrinogen activators, matrix metalloproteinases, and lysosomal peptidases. It has been shown 81 that APOE promotes the degradation of Aβ in microglia in an isoform-dependent manner via neprilysin (NEP) and insulin-degrading enzyme (IDE) in the extracellular space. Enhanced expression of lipidated APOE also stimulates protein-hydrolyzed Aβ degradation via living X receptors (LXRs) and ABCA1. 76

### Apolipoprotein E and tau

The effect of APOE on tau is also isomer-dependent. Compared with APOE2 and APOE3, APOE4 can increase tau phosphorylation when Aβ oligomers are present. Although the number of clinical studies on the effects of APOE genotypes on tau protein is limited, the Harris et al. 82 have shown that APOE4 increases total tau and phosphorylated tau levels in pathological models of tau hyperphosphorylation. Moreover, APOE4 can exacerbate tau-mediated neurodegeneration by regulating the activation of microglia, which is consistent with the findings of a previous neuropathological study. 83 Another study 81 showed that selective knockout of APOE4 in astrocytes reduced tau-related synaptic loss and inhibited microglial phagocytosis of synapses.

Another study 85 using a tau delivery method with an adeno-associated virus (AAV) revealed that APOE2 may lead to increased tau phosphorylation and aggregation, which is associated with an increased risk of developing primary tauopathies. And the increase in tau aggregation may be due to the formation of tau/APOE complexes, which occurs predominantly in the presence of nonliposomalized APOE2. Recently, the Genome Wide Association Study 86 (GWAS) revealed that APOE2 significantly regulates the activity of protein phosphatase 2A (PP2A), the major tau phosphatase in the human brain, which prevents AD risk. The authors 86 further suggest that the protective mechanism of APOE2 may differ from the deleterious effects of APOE4 on the risk of cognitive impairment. These results demonstrate the role of APOE in tau-mediated neurotoxicity, which provide supporting evidence that the effects of APOE are isoform-dependent.

Moreover, APOE has been shown 87 to directly bind to the tau protein and block its phosphorylation sites, and this binding interaction is also isomer-dependent. The binding affinity of APOE3 for the microtubule-binding region of tau is much stronger than that of APOE4, 88 and the reduced binding affinity of APOE4 for tau can increase tau hyperphosphorylation mediated by GSK3, leading to an increase in the formation of NFTs. 89 Moreover, APOE4 increases GSK3 activity by preventing Wnt signaling via the LRP5/6 receptor, resulting in increased tau phosphorylation. 90 However, the specific mechanism needs more in-depth research. In brief, although these effects need further clarification, especially the impact of different APOE genotypes on tau hyperphosphorylation, these findings indicate that APOE dysfunction affects tauopathies in the brain.

### Apolipoprotein E and insulin resistance

Although both APOE4 and non-APOE4 carriers with AD have low insulin concentrations in cerebrospinal fluid (CSF) and evidence of brain insulin resistance, APOE4 carriers with AD show a worse response to intranasal insulin treatment than do AD patients who do not carry APOE4, and this phenomenon can be observed not only with fast-acting insulin but also with short-acting insulin. 91 Interestingly, long-acting insulin had dual ameliorative effects on cognitive ability and peripheral insulin resistance in APOE4 carriers. 91 This finding 91 may be related to the fact that APOE4 carriers have a greater degree of insulin resistance in the brain and require long-acting insulin agents to alter brain insulin metabolism. Other studies 92 93 have indicated that the relationship between Aβ and insulin resistance is also influenced by APOE4 status; for example, an epidemiological study 92 reported that only non-APOE4 carriers showed a relationship between the Aβ concentration in CSF and the ratio of CSF to plasma glucose, and only this group showed an improvement in memory and a decrease in plasma levels of APP after insulin infusion, whereas the memory of APOE4 carriers remained unchanged, and APP plasma levels actually increased in response to insulin. 93 Moreover, IR is widely distributed in the brain, and the APOE4 allele can act on IR, to block its transport from the nucleus to the cell membrane, leading to insulin signal transduction disorders. Meanwhile, peripheral insulin resistance and AOPE4 synergistically impair insulin signaling, and APOE4 could reduce insulin-IR interaction and impair IR trafficking; compared with APOE3 carriers, the APOE4 carriers showed a reduction in the phosphorylation status of the Akt (Ser473) and GSK3B (Ser9)IRSs. 94 In conclusion, these studies 91 92 93 94 identified differences between APOE4 carriers and non-APOE4 carriers in the regulation by central insulin of cognitive processes, memory, and peripheral insulin and other hormone metabolism . 95 Additionally, APOE4 can impair the insulin signaling pathway in the mouse brain and neurons; nevertheless, APOE2 can reverse these impairments. Moreover, a recent study 96 showed that APOE4 can directly interfere with IRs to mediate the aforementioned processes. Taken together, these results suggest that disturbances in brain glucose metabolism and/or insulin signaling induced by APOE isoforms may be key to understanding the mechanisms of neurodegenerative changes in individuals with cognitive impairment.

## CONCLUSIONS, CHALLENGES IN TARGETING THE COMPLEMENT, AND FUTURE PERSPECTIVES

Lipids are important materials to maintain the structure of cell membranes and regulate intracellular neurotransmitter synthesis and release, as well as synaptic growth. Disruption of these biological processes plays a crucial role in the pathogenesis of cognitive impairment associated with AD, DCI, and VD. In fact, the progression of these three common types of cognitive impairment largely depends on lipid metabolism abnormalities. Given the coordinated effects of the three pathological mechanisms of Aβ deposition, tau hyperphosphorylation, and insulin resistance on the development of cognitive disorders, fully exploring the role of lipid metabolism and its underlying pathological mechanisms in the pathogenesis of AD, DCI, and VD will help us to target lipid metabolism to develop therapeutic strategies. In the present review, we comprehensively explored the relationships between lipid metabolism and the most common types of cognitive disorders (AD, DCI, and VD), as well as the important role of lipid metabolic disorders in their common pathogenic mechanisms. These findings indicate that:

- The progression of cognitive impairment associated with AD, DCI, and VD is in part due to lipid metabolic disorders among various cells in the brain microenvironment;
- Lipid metabolic disorders in patients with cognitive impairment associated with AD, DCI, and VD are closely related to their common pathogenic mechanisms (Aβ deposition, tau hyperphosphorylation, and insulin resistance);
- As an important protein for lipid transport, APOE is closely related to the common pathogenesis of AD, DCI, and VD, and there is heterogeneity in the regulatory effects of APOE on these three pathogenic mechanisms (Aβ deposition, tau hyperphosphorylation, and insulin resistance).

For example, the composition of the neuronal membrane structure, the synthesis and release of neurotransmitters, and the generation and extension of synapses all involve lipid metabolism. Lipid metabolic disorders can affect neural signal transduction by regulating these processes, which can negatively impact the cognitive function of patients. Lipid metabolic disorders can accelerate Aβ deposition, tau protein hyperphosphorylation, and insulin resistance to form a vicious cycle, ultimately exacerbating the progression of cognitive impairment. Moreover, APOE4, an isomer of APOE, is more likely to aggravate Aβ deposition, tau protein hyperphosphorylation, and insulin resistance than APOE2 and APOE3. Therefore, these factors collectively determine the impact of lipid metabolism on cognitive impairment associated with AD, DCI, and VD, ultimately accelerating disease progression.

Drugs targeting metabolic dysfunction are the current focus of cognitive dysfunction research; however, strategies targeting lipid metabolism for the treatment of cognitive impairment are still in the exploratory stage, and relevant high-level evidence-based medical evidence supporting this strategy is lacking. The reasons may include the complexity of lipid metabolism and the diversity of lipid metabolic regulation in the pathogenesis of cognitive impairment associated with AD, DCI, and VD. Therefore, combining the aforementioned findings and previous reports, we suggest topics that need to be addressed regarding lipid metabolism in individuals with cognitive impairment: the complex mechanisms of lipid metabolism regulation in individuals with cognitive impairment still need further exploration, such as the important role of key enzymes in lipid metabolic synthesis, catabolism, and transport processes in the pathogenesis of cognitive impairment.

Given that the key protein for lipid transport, APOE, has different isomers and different roles in lipid metabolism, research on APOE as a therapeutic target for cognitive impairment should focus on assessing the impact of different isomers of APOE on disease progression to determine the key subtypes that induce the progression of cognitive impairment and to provide reliable targets for the treatment of cognitive impairment. In general, a more in-depth study of lipid metabolic abnormalities in cognitive impairment processes such as AD, DCI, and VD, as well as lipid metabolic crosstalk between different cells in the brain microenvironment, will help us fully understand the relevant mechanisms of cognitive impairment, which is crucial to fully target lipid metabolism to treat the cognitive impairment associated with AD, DCI, and VD.

In summary, lipid metabolism is important for the treatment of cognitive disorders. The present study not only helps to reveal the underlying mechanisms of cognitive disorders and provide novel therapeutic targets but also promotes early warning and diagnosis as well as interdisciplinary research and cooperation. In the future, with the deepening of research, we believe that targeting lipid metabolism will become an important breakthrough in the treatment of cognitive disorders.
