# Erratum

**DOI:** 10.1055/s-0045-1806749

**Published:** 2025-03-19

In the article “The role of lipid metabolism in cognitive impairment”, published in Arq. Neuro-Psiquiatr. 2025;83(01):s00441792097 under DOI 10.1055/s-0044-1792097 .

Where it reads:

^1^ Second Medical University, School of Clinical Medicine, Weifang Shandong Province, China.

It should be:

^1^ Shandong Second Medical University, School of Clinical Medicine, Weifang Shandong Province, China.

